# Effects of rTMS Intervention on Functional Neuroimaging Activities in Adolescents with Major Depressive Disorder Measured Using Resting-State fMRI

**DOI:** 10.3390/bioengineering10121374

**Published:** 2023-11-29

**Authors:** Fengmei Lu, Qian Cui, Yang Zou, Yuanhong Guo, Wei Luo, Yue Yu, Jingjing Gao, Xiao Cai, Linna Fu, Shuai Yuan, Juan Huang, Yajun Zhang, Jing Xie, Wei Sheng, Qin Tang, Qing Gao, Zongling He, Huafu Chen

**Affiliations:** 1 The Clinical Hospital of Chengdu Brain Science Institute, School of Life Science and Technology, University of Electronic Science and Technology of China, Chengdu 611731, China; fengmei_lu@126.com (F.L.); zouyang2023@126.com (Y.Z.); gyhnice@163.com (Y.G.); lwlw567@163.com (W.L.); yu8382508@163.com (Y.Y.); 13568944575@163.com (X.C.); fulinna1207@163.com (L.F.); hkdyuanshuai@163.com (S.Y.); 18715797964@139.com (J.H.); 13509878261@163.com (Y.Z.); 13622582173@163.com (J.X.); acca.swei@gmail.com (W.S.); tangqin0201@163.com (Q.T.); 2 School of Public Affairs and Administration, University of Electronic Science and Technology of China, Chengdu 611731, China; 3 School of Information and Communication Engineering, University of Electronic Science and Technology of China, Chengdu 611731, China; jingjing.gao@uestc.edu.cn; 4 School of Mathematical Sciences, University of Electronic Science and Technology of China, Chengdu 611731, China; 5 MOE Key Lab for Neuroinformation, University of Electronic Science and Technology of China, Chengdu 611731, China; 6 High-Field Magnetic Resonance Brain Imaging Key Laboratory of Sichuan Province, University of Electronic Science and Technology of China, Chengdu 611731, China

**Keywords:** repetitive transcranial magnetic stimulation, adolescent, major depressive disorder, neuroimaging biomarkers

## Abstract

Repetitive transcranial magnetic stimulation (rTMS) to the left dorsolateral prefrontal cortex (L-DLPFC) is commonly used for the clinical treatment of major depressive disorder (MDD). The neuroimaging biomarkers and mechanisms of rTMS are still not completely understood. This study aimed to explore the functional neuroimaging changes induced by rTMS in adolescents with MDD. A total of ten sessions of rTMS were administrated to the L-DLPFC in thirteen adolescents with MDD once a day for two weeks. All of them were scanned using resting-state functional magnetic resonance imaging at baseline and after rTMS treatment. The regional homogeneity (ReHo), amplitude of low-frequency fluctuation (ALFF), and the subgenual anterior cingulate cortex (sgACC)-based functional connectivity (FC) were computed as neuroimaging indicators. The correlation between changes in the sgACC-based FC and the improvement in depressive symptoms was also analyzed. After rTMS treatment, ReHo and ALFF were significantly increased in the L-DLPFC, the left medial prefrontal cortex, bilateral medial orbital frontal cortex, and the left ACC. ReHo and ALFF decreased mainly in the left middle occipital gyrus, the right middle cingulate cortex (MCC), bilateral calcarine, the left cuneus, and the left superior occipital gyrus. Furthermore, the FCs between the left sgACC and the L-DLPFC, the right IFGoper, the left MCC, the left precuneus, bilateral post-central gyrus, the left supplementary motor area, and the left superior marginal gyrus were enhanced after rTMS treatment. Moreover, the changes in the left sgACC-left MCC FC were associated with an improvement in depressive symptoms in early improvers. This study showed that rTMS treatment in adolescents with MDD causes changes in brain activities and sgACC-based FC, which may provide basic neural biomarkers for rTMS clinical trials.

## 1. Introduction

Major depressive disorder (MDD) is one of the most common serious mental illnesses and the second leading cause of mental disability in China; it affects millions of people worldwide [1,2,3]. MDD in adolescents has a lifetime prevalence as high as 14% to 20%, being a major cause of morbidity and disability, severely impinging on various areas of daily functioning in adolescent individuals [4,5]. Therefore, improving the treatment of adolescents with MDD is important for improving public health. The preferred treatments for adolescents with MDD include pharmacotherapy such as antidepressants, psychosocial interventions such as psychotherapy, or combinations of these [6,7]. Unfortunately, about 38~52% of adolescents with MDD do not respond adequately to these traditional treatments [6]. There still remains concern and controversy about the safety and effectiveness of antidepressants in adolescents with MDD [8,9]. Some antidepressants may even lead to a variety of side effects, such as suicidal ideation and behavior [8,10,11]. These findings suggest an urgent need for alternative effective interventions for adolescents with MDD.

Over the past few decades, brain stimulation methods have become increasingly recognized as effective treatments for patients with MDD, in addition to medication and psychotherapy [12]. Among these, repetitive transcranial magnetic stimulation (rTMS), a noninvasive brain neuromodulation technique approved by the US Food and Drug Administration in 2008, is demonstrated to be an efficacious intervention for MDD patients [13,14,15]. However, studies also indicate that not all MDD patients showed sufficient rTMS responsiveness, the effective rate is about 55% and varies greatly among MDD patients [16,17,18]. Thus, to advance rTMS in treating adolescents with MDD, it is important to recognize its impact on adolescent neurobiology. This understanding may help to find pretreatment biomarkers to identify patients who may effectively respond to rTMS.

Resting-state functional magnetic resonance imaging (fMRI)—a non-invasive, safe, and reliable repeated measurement—has become an effective and popular method of studying the human brain [19,20], for which neuroimaging measurements have been recognized as potential biomarkers [21]. Among these, the regional homogeneity (ReHo) and the amplitude of low-frequency fluctuation (ALFF) are two basic fMRI parameters with a high test-retest reliability. The ReHo measures the similarity between the time series of a given voxel and its nearest voxel, representing the synchronization of neuronal activities in the local brain regions [22], while ALFF reflects spontaneous fluctuations in the low-frequency range of blood-oxygen-level-dependent (BOLD) signals, which are closely related to neuronal activities [23]. Furthermore, studies have demonstrated that the intrinsic resting-state functional connectivity (FC) between the subgenual anterior cingulate cortex (sgACC) and other brain regions was associated with the therapeutic response of rTMS for treating MDD [24,25,26,27]. The sgACC was involved in emotional processing and was a deep brain stimulation (DBS) target for MDD [28,29,30]. Therefore, we speculated that rTMS could impact adolescents with MDD by affecting the FC between the sgACC and other brain regions. Overall, this study recruited adolescents with MDD, who received a 10 Hz rTMS intervention for two weeks. We aimed to investigate the functional neuroimaging effects of rTMS treatment on adolescents with MDD, and to examine whether the longitudinal changes of sgACC-based FC were correlated with changes in clinical symptoms.

## 2. Materials and Methods

### 2.1. Subjects

A total of thirteen right-handed adolescent MDD patients (age range 12~21 years old) were recruited from the Clinical Hospital of Chengdu Brain Science Institute, University of Electronic Science and Technology of China. All patients were interviewed by two experienced psychiatrists with the Structured Clinical Interview for DSM-IV-TR—Patient Edition (SCID-P, 2/2001 revision). Patients were excluded if they met any of the criteria as follows: (1) a diagnosis of bipolar disorder, anxiety disorder, obsessive compulsive disorder, conduct disorder, attention deficit/hyperactivity disorder, post-traumatic stress disorder, or schizophrenia; (2) had a past or current neurological disorder such as stroke, head injury, dementia or other neurodegenerative disorder, history of seizures, history of cranial trauma, and brain surgery; (3) had a history of alcohol abuse, substance abuse, or drug use; (4) had cardiac pacemakers, intracranial implant, or ferromagnetic parts in the cranium that are not suitable for TMS; (5) had a history of brain stimulation treatment (TMS, electroconvulsive therapy, or transcranial direct current stimulation) in the last 3 month. Written informed consent was obtained by all the participants or their parents. The current study was approved by the Ethics Committee of the Clinical Hospital of Chengdu Brain Science Institute, University of Electronic Science and Technology of China, was conducted in line with the latest version of Declaration of Helsinki, and was registered at ClinicalTrials.gov (Identifier: NCT02888509).

### 2.2. rTMS Procedure

All patients underwent the rTMS treatment with a figure-of-eight shaped coil using an YRD CCY-I magnetic simulator (YIRUIDE Inc., Wuhan, China) [31,32,33]. In detail, a total of ten sessions on the left dorsolateral prefrontal cortex (L-DLPFC) were delivered on the patients once a day on weekdays for two weeks. Prior to the first treatment, the study physician determined the individual stimulus intensity according to the resting motor threshold (RMT), which is defined as the minimum stimulus intensity that generates a motor-evoked potential of greater than 50 μV in 50% of 10 trials [34,35]. Stimulation was applied to the L-DLPFC at 110% RMT and a 10 Hz frequency. Stimulus trains were 5 s with an inter-train interval of 55 s; the treatment session lasted for 20 minutes and consisted of 1000 pulses. The stimulation position for the L-DLPFC was localized using the standard 5 cm rule as described in previous studies [32,36,37,38]. All patients were taking medication and they were on stable antidepressant regimes during the 2-week rTMS treatment period.

### 2.3. Clinical Assessment

Patients’ depression and anxiety severity were measured by using the Hamilton Depression Rating Scale (HAMD-24) [39] and the 14-item Hamilton Anxiety Scale (HAMA) at two time points prior to the MRI scan: within 1~2 days before the first rTMS treatment session (pre-rTMS) and within 1~2 days after the last rTMS treatment session (post-rTMS). Information about their age of first onset, number of depressive episodes, and duration of single episode were also collected. The improvement ratio in depressive and anxiety symptoms was measured by the value of the pre-rTMS scores minus post-rTMS scores in HAMD and HAMA divided by the pre-rTMS scores.

### 2.4. fMRI Data Acquisition

The resting-state fMRI data were acquired with a 3.0-Tesla GE Discovery MR750 scanner (General Electric, Fairfield, CT, USA) located at the University of Electronic Science and Technology of China at two time points: within 1~2 days before the first rTMS treatment session and within 1~2 days after the last rTMS treatment session. Before the scan, we first asked each participant if they had claustrophobia, brain trauma, or other MRI contraindications such as metal implants. Secondly, we explained the whole process of the experiment in detail, including how long the MRI scan would take, and foam pads and soft earplugs were utilized to reduce head movement and the machine noise. Finally, we let each participant observe the internal environment of the MRI scanner and let them lie in the scanner for a few minutes so that they could adapt to the MRI environment in advance. Then, they signed the informed consent form and could withdraw from the scan at any time if they felt uncomfortable. All the participants were instructed to keep still, close their eyes, and relax while remaining awake. A gradient-recalled echo-planar imaging (EPI) sequence was used as follows: repetition time, 2000 ms; echo time, 30 ms; matrix size, 64 × 64; field of view, 240 mm × 240 mm; voxel size, 3.75 mm × 3.75 mm × 3.2 mm; flip angle, 90°; slice thickness, 3.2 mm; no gap, and 43 axial slices. A total of 255 volumes were collected and the scanning time was 510 s.

### 2.5. fMRI Data Preprocessing

fMRI data were preprocessed using the Data Processing Assistant for Resting-State fMRI (Advanced Edition, DPARSFA, v4.5) software of the Data Processing and Analysis of Brain Imaging (DPABI) toolkit (http://rfmri.org/dpabi (accessed on 25 July 2019)). In brief, the following processing steps were applied: discarding the first 15 volumes, slice-timing correction, head motion correction by realignment, spatial normalization using the standard EPI Montreal Neurological Institute (MNI) template at a resolution of 3 × 3 × 3 mm^3^, spatially smoothed with a Gaussian kernel of 6 mm full-width at half-maximum (FWHM), and linear detrending and temporal bandpass filtering (0.01~0.1 Hz). Data were excluded where translational or rotational motion parameters exceeded ± 3.0 mm or ± 3.0° [3,40,41]. The subject’s mean frame-wise displacement (FD) was calculated [23,42,43,44]. Several nuisance covariates including 24 motion parameters, averaged signals from the white matter, cerebrospinal fluid, and global signals were regressed out [45,46]. Finally, a “scrubbing” method at an FD threshold of 0.5 mm was utilized to reduce the impact of motion artifacts; the “bad” time points were identified and estimated using cubic spline interpolation [47,48,49].

### 2.6. ReHo and ALFF Calculation

In this study, two indexes including ReHo [22] and ALFF [19] were calculated in patients before and after treatment. Specifically, for ReHo, Kendall’s coefficient of concordance of each voxel was calculated with its nearest 26 voxels to obtain the individual ReHo map. For ALFF, the filtered time series of each voxel was converted to the frequency domain using fast Fourier transform. The square root of the power was calculated at each frequency to obtain amplitude values in the low-frequency range of 0.01 to 0.1 Hz, then the averaged square root was obtained. ALFF was computed as the sum of the amplitude values. Next, for standardization, the resulting ReHo and ALFF maps were converted into z-score maps by subtracting the mean value of each voxel and dividing by the standard deviation. Finally, the standardized ReHo maps were smoothed with a Gaussian kernel of 6 mm FWHM.

### 2.7. sgACC-Based Whole-Brain FC Analysis

The left sgACC (DBS target) was selected as the region of interest (ROI) which is located ipsilateral to the L-DLPFC (stimulated rTMS target) to minimize the bias of the bilateral sgACC. The MNI coordinates of left sgACC was −10, 35, and −2 based on the previous study [50] and we draw a sphere with 3 mm radius to create the seed region. Then, we computed the FC between the left sgACC and the whole brain in patients before and after treatment. Standardized seed-based FC analysis was conducted using the Resting-State fMRI Data Analysis Toolkit (REST, http://restfmri.net/forum/REST_V1.8 (accessed on 25 July 2019)). In brief, the Pearson’s correlation coefficients were calculated between the time series of the left sgACC and all the other remaining voxels in the whole brain. Ultimately, Fisher’s r-to-z transformation method was utilized to convert the whole-brain FC maps into z maps for further statistics analyses. 

### 2.8. Statistical Analysis

Paired-sample *t*-tests were used to compare the HAMD scores and HAMA scores of MDD patients before and after treatment using with Statistical Package for the Social Sciences (SPSS; IBM SPSS Statistics for Windows, Version 26.0, IBM Corp., Armonk, NY, USA). The significance level was set at *p* < 0.05.

To examine the effects of two-week rTMS treatment on ReHo, ALFF and sgACC-based FC, paired-sample *t*-tests were conducted between MDD patients before and after treatment. The statistical significance was set at *p* < 0.05 (AlphaSim corrected, combined height threshold *p* < 0.05 and a minimum cluster size of 925 voxels for ReHo, 508 voxels for ALFF, and 453 voxels for sgACC-based FC). The AlphaSim correction was implemented in the AlphaSim program using Monte Carlo simulation with the REST software (http://www.restfmri.net (accessed on 25 July 2019)) [51]. 

Further, to explore whether the changes in depressive symptoms were associated with the impacts of two-week rTMS treatment on sgACC-based FC, Pearson’s correlation analysis was applied between symptom improvement and sgACC-based FC alterations. We also analyzed the relationships between the symptom improvement and sgACC-based FC alterations in early improvers (whose HAMD scores were reduced by at least 20% from pre-rTMS to post-rTMS). The reduction ratio of symptom improvement was calculated as the %HAMD scores reduction = (HAMD scores_pre-rTMS_ − HAMD scores_post-rTMS_)/HAMD scores_pre-rTMS_ × 100%. The sgACC-based FC changes were computed as ΔzFC = zFC_post-rTMS_ − zFC_pre-rTMS_. The statistical significance was defined at *p* < 0.05.

## 3. Results

### 3.1. Demographic and Clinical Outcomes

Two patients were excluded due to data quality including one patient before treatment and another patient after treatment. Demographic characteristics in terms of the age, gender, educational level, and mean FD of the final participants are shown in Table 1. The two subgroups (pre-rTMS and post-rTMS) did not differ significantly in mean FD. After two weeks of rTMS intervention, the total HAMD scores and HAMA scores were significantly reduced (Table 1).

### 3.2. rTMS-Induced ReHo Changes between Pre-rTMS and Post-rTMS

**Figure 1:**
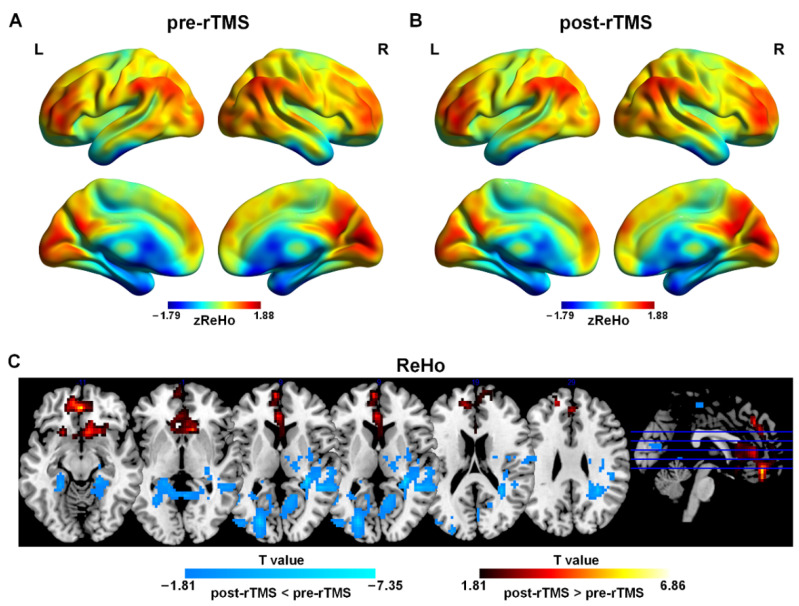
Spatial distribution patterns of ReHo in adolescents with MDD pre-rTMS (**A**) and post-rTMS (**B**) treatment. Warm colors represent that ReHo was higher than the whole brain level, while cool colors show that ReHo was lower than the whole brain level (*p* < 0.001, FDR correction). (**C**) ReHo differences in adolescents with MDD pre-rTMS and post-rTMS. Warm colors represent that the values were higher in post-rTMS than those in pre-rTMS, while cool colors show that the values were lower in post-rTMS than those in pre-rTMS (*p* < 0.05, AlphaSim correction).

The one-sample *t*-test results showed that patients before and after treatment had typical distribution patterns of ReHo (Figure 1A,B). Brain regions with higher ReHo were mainly located in the prefrontal cortex (PFC), the posterior cingulate gyrus (PCC)/precuneus (PCUN), the medial prefrontal cortex (mPFC), and bilateral angular gyrus and other brain regions including the supplementary motor area (SMA), the pre-central gyrus (PreCG), the post-central gyrus (PoCG), and the calcarine (CAL).

The paired-sample *t*-tests results demonstrated that after rTMS treatment, ReHo in the bilateral medial orbital frontal cortex (mOFC), the left mPFC, the left anterior cingulate gyrus (ACC), and the left gyrus rectus was higher than that in pre-rTMS. ReHo in the left middle occipital gyrus (MOG), the right middle cingulate cortex (MCC), the bilateral CAL and the left cuneus (CUN) was lower than that in pre-rTMS (Figure 1C and Table 2).

### 3.3. rTMS-Induced ALFF Changes between Pre-rTMS and Post-rTMS

**Figure 2:**
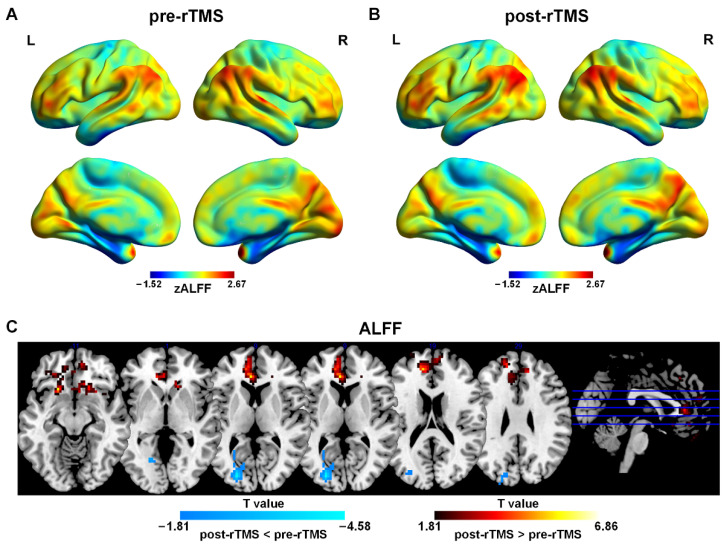
Spatial distribution patterns of ALFF in adolescents with MDD pre-rTMS (**A**) and post-rTMS (**B**) treatment. Warm colors represent that ALFF was higher than the whole brain level, while cool colors show that ReHo was lower than the whole brain level (*p* < 0.001, FDR correction). (**C**) ALFF differences in adolescents with MDD pre-rTMS and post-rTMS. Warm colors represent that the values were higher in post-rTMS than those in pre-rTMS, while cool colors show that the values were lower in post-rTMS than those in pre-rTMS (*p* < 0.05, AlphaSim correction).

The one-sample *t*-test results showed that patients before and after treatment have typical distribution patterns of ALFF (Figure 2A,B). Brain regions with higher ALFF were mainly located in the PFC, the PCC/PCUN, the mPFC, and bilateral angular gyrus and other brain regions including the CUN, the superior temporal gyrus, the supplementary motor area (SMA), and the CAL.

The paired-sample *t*-test results indicated that after rTMS treatment, ALFF was higher in the left DLPFC, the left ACC, and the left mPFC than that in pre-rTMS. ALFF in the left MOG and the left SOG was lower than that in pre-rTMS (Figure 2C and Table 2).

### 3.4. rTMS-Induced FC Changes between Pre-rTMS and Post-rTMS

**Figure 3:**
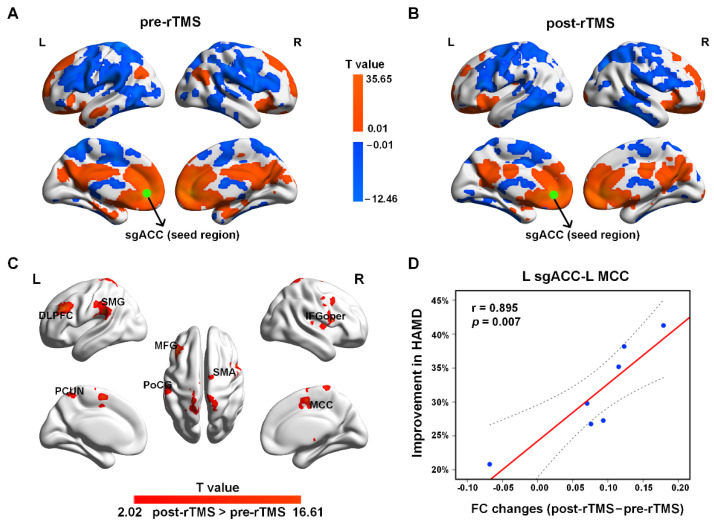
The sgACC-based FC patterns in adolescents with MDD pre-rTMS (**A**) and post-rTMS (**B**) treatment. The green dots represent the seed of sgACC. (**C**) Increased FC after rTMS treatment (*p* < 0.05, AlphaSim corrected). (**D**) The increased FC between the left sgACC and left MCC was positively associated with the ratio of HAMD improvement in early improvers (including seven MDD patients) (*p* < 0.05). The dotted line represents the 95% confidence interval.

The one-sample *t*-test results showed that the brain areas showing negative FC with the left sgACC were mainly in the DLPFC, the inferior frontal gyrus (IFG), the PreCG, the PoCG, the temporal lobe, the SMA, the fusiform gyrus, the putamen, the caudate, the insula, and the thalamus (Figure 3A,B). The areas showing positive FC with the left sgACC were mainly in the mPFC, the mOFC, the posterior cingulate gyrus/PCUN, the superior and inferior frontal gyrus, the amygdala, the MCC, the CUN, the hippocampus, the CAL, and the parahippocampal gyrus, as well as the middle temporal gyrus (Figure 3A,B).

The paired-sample *t*-test results suggested that after treatment, the FC was increased mainly between the left sgACC and the left DLPFC, the left MCC, the middle frontal gyrus (MFG), the IFG, the opercular part (IFGoper), the PCUN, the PoCG, the SMA, and the superior marginal gyrus (SMG) (Figure 3C and Table 3). No decreased FC in the patients was found after rTMS treatment. In addition, after rTMS treatment, the increased FC between the left sgACC and the left MCC in early improvers (including seven MDD patients) was positively correlated with the ratio of HAMD improvement (Figure 3D). No other significant relationships were found between symptom improvement and sgACC-based FC alterations. 

## 4. Discussion

The current study indicated the efficacy and safety of rTMS in treating adolescents with MDD, and explored the therapeutic neural mechanisms of rTMS intervention by using ReHo and ALFF, as well as the sgACC-based FC analysis. After rTMS treatment, ReHo and ALFF were increased predominantly in the frontal cortex (the left mPFC, bilateral mOFC, the left DLPFC, and the left gyrus rectus) and the left ACC, while ReHo and ALFF were reduced mainly in the left MOG, the right MCC, bilateral CAL, the left CUN, and the left SOG. In addition, the sgACC-based FC was enhanced between the left sgACC and the left DLPFC, the right IFGoper, the left MCC, the left PCUN, bilateral PoCG, the left SMA, and the left SMG in adolescents with MDD after treatment. Furthermore, there was a positive association between the increased FC of the left sgACC–left MCC and the reduction ratio of HAMD scores in early improvers, suggesting that the stronger FC of the left sgACC–left MCC was associated with more clinical improvement after two-week rTMS treatment. These findings demonstrated the treatment effects of rTMS on the ReHo, ALFF, and FC in various regions, and may help us to further understand the neural mechanisms of rTMS in the treatment of adolescents with MDD.

Importantly, after rTMS treatment, ReHo and ALFF were significantly enhanced in the left DLPFC, the left mPFC, and bilateral mOFC, which were the target region of rTMS as well as nearby frontal regions. Meanwhile, ReHo and ALFF in the left ACC were also increased. Depression is increasingly identified as a network disorder related with abnormalities in widely spread brain regions including the PFC such as the DLPFC (particularly the left), the mPFC, the OFC, and limbic systems such as the ACC (especially the subcallosal ACC such as the sgACC), the insula, the thalamus, and the hippocampus, and so on [24,52,53]. The PFC is a pivotal region implicated in a wide range of functions including executive function, decision-making, emotional regulation, and reward-guided behavior, which is involved in the neurobiological mechanisms underlying MDD [54,55]. Previous evidence has indicated that the brain activity in the PFC is decreased in MDD patients [56,57,58,59]. The ACC is also involved in the top-down control of emotions that is related with the processing of rewards and non-rewards, and also implicated in various functions such as social processing, error and conflict monitoring, and decision-making [60,61,62,63]. The rTMS applies focal magnetic stimulation to the brain scalp and induces electrical stimulation in cortical tissue, which could change cortical activity and modulate brain regions and networks [64]. Numerous studies have demonstrated that the left DLPFC is one of the most typically employed target site of rTMS for MDD, and high-frequency stimulation (>5 Hz) has excitatory effects which leads to cortical excitability in the brain [32,33]. Moreover, a random effects meta-analysis containing 30 double-blind sham-controlled clinical trials has demonstrated that 10 Hz rTMS over the left DLPFC could increase the neural activity and ameliorate depressive symptoms for MDD [17]. Our findings were consistent with previous studies and provide further evidence that high-frequency rTMS enhanced the excitability of neurons.

Furthermore, the FC between the left sgACC and the left DLPFC, the right IFGoper, the left MCC, and the left PCUN was enhanced after rTMS treatment. The sgACC is involved in modulating and processing emotional stimulus and behavior output and is usually applied as a probe to examine emotional networks in MDD patients [50,65]. sgACC has been widely utilized as a stimulating target in treating MDD [66,67,68]. Studies have shown decreased FCs between the sgACC and the insula, and the PFC regions (the inferior and superior frontal gyrus) in adolescents with MDD as well as in children with preschool-onset MDD [69,70]. What is more, a prior study also documented reduced FC between the sgACC and the PCUN in adolescents with MDD [71]. The MCC plays a key role in emotional regulation, emotional feedback integration, decision-making, social cognition, and negative affect [72,73,74], which is linked with the physiopathology of depression. In addition, we found that the stronger left sgACC–left MCC FC was related with more improvement in depressive symptoms in early improvers. After treatment, the FCs in the sgACC were increased, suggesting that the enhancement of FCs in the sgACC may have a protective effect on depression and a positive effect on the treatment of depression. A previous study confirmed that the FC between the sgACC and the left DLPFC could predict the rTMS efficacy [25]. Our findings may also support that the sgACC FC patterns with deeper brain regions might predict the efficacy of rTMS in treating MDD, and strengthen the role of the sgACC in the pathophysiology of MDD and as a therapeutic target. 

This study has some limitations that should be considered. First, the sample size was small and most of the participants were female. Previous epidemiological studies have indicated that females were twice as likely as males to present a depressive episode [75,76]. Future studies should be conducted with a larger sample size and a relatively balanced proportion of male and female adolescents with MDD. Second, there was no control group, neither HCs nor a sham control group. In the future, a randomized double-blinded clinical study should be performed to explore the changes at baseline between patients and HCs, and to investigate the changes between pre-rTMS and post-rTMS, as well as to examine the effects of sham rTMS versus active rTMS. Third, the patients in our study were a real-world sample who have also taken medications during the rTMS treatment period. However, we tried to select a group of patients on monotherapy with one type of antidepressant medication and make sure that they maintained a stable dose during rTMS treatment sessions. Future studies should also take the medication information as a variable of interest in the analysis. Furthermore, some recent studies have shown an association between electroencephalography (EEG) powers and FC with rTMS treatment outcomes in MDD, suggesting more clinically translatable techniques. For example, one study demonstrated that the power characteristics of resting-state EEG (rsEEG) could effectively predict the improvement of symptoms of MDD patients with sertraline, and the sertraline predicted EEG signature was related differentially to the rTMS treatment outcome [77]. Another study which identified two MDD subtypes from rsEEG FC patterns showed that both subtype 1 and subtype 2 MDD patients responded similarly well to two different rTMS regimens (left DLPFC 10 Hz and right DLPFC 1 Hz, both accompanied by psychotherapy) [78]. Therefore, in the future, EEG brain measurement could also be applied to explore the rsEEG FC for rTMS treatment responsive biomarkers. Finally, studies have also shown that the frontoparietal control network and default mode network FC could also serve as a general and differential biomarker for predicting treatment outcomes [78,79]. Thus, it would also be interesting to examine the typical regions in these networks in future studies. 

## 5. Conclusions

In conclusion, our results showed that a two-week rTMS intervention in adolescents with MDD was associated with changes in brain neuroimaging activities and sgACC-based FC, and FC change in sgACC-MCC was related with an improvement in depressive symptoms in early improvers. The findings may provide basic neural biomarkers and substrates for rTMS clinical trials. From the perspective of mechanism, the current results of ReHo, ALFF, and FC changes in adolescents with MDD before and after treatment may help us to better understand the neural mechanisms associated with rTMS treatment response at the local brain activity level and the network level. On the other hand, from a clinical point of view, our findings of a stronger sgACC-MCC FC associated with more clinical improvement may help to identify individuals who respond well to rTMS and may provide useful prognostic information.

## Figures and Tables

**Table 1:** Characteristics of patients between pre-rTMS and post-rTMS.

Characteristic	pre-rTMS (*n* = 11)	post-rTMS (*n* = 11)	t/F	*p*
Age (years)	16.18 ± 2.36	16.18 ± 2.36		
Education (years)	10.05 ± 2.39	10.05 ± 2.39		
Gender (M:F)	1:10	1:10		
Age of first onset (years)	13.73 ± 1.95	13.73 ± 1.95		
Number of depressive episodes	1.64 ± 1.21	1.64 ± 1.21		
Duration of single depressive episode (months)	10.82 ± 9.53	10.82 ± 9.53		
Mean FD (mm)	0.09 ± 0.03	0.08 ± 0.04	0.78	0.453 *
HAMD score	51.55 ± 16.59	36.73 ± 11.31	26.04	<0.001 ^#^
HAMA score	31.09 ± 9.76	21.55 ± 5.35	24.17	0.001 ^#^

M, male; F, female; FD, frame-wise displacement; HAMD, Hamilton Rating Scale for Depression; HAMA, Hamilton Anxiety Rating Scale. The values are represented as the mean ± SD. * The *p* value is obtained by paired-sample *t*-tests. ^#^ The *p* value is obtained by a one-way repeated measures analysis of variance (ANOVA).

**Table 2:** Comparison of ALFF and ReHo between pre-rTMS and post-rTMS.

Brain Region	MNI Coordinates			Cluster Size	T-Value
	x	y	z	(Voxels)	
ReHo					
post-rTMS > pre-rTMS					
Rectus_L	−3	45	−15	84	6.86
mPFC_L	−3	36	39	167	5.21
mOFC_L	−3	45	−12	96	5.03
mOFC_R	9	42	−6	77	3.61
ACC_L	−3	36	6	103	3.81
post-rTMS < pre-rTMS					
MOG_L	−24	−81	12	161	−4.54
CUN_L	−21	−78	12	150	−7.35
MCC_R	21	−15	63	94	−4.42
CAL_L	−18	−75	12	114	−3.69
CAL_R	9	−60	9	126	−3.45
ALFF					
post-rTMS > pre-rTMS					
mPFC_L	−12	45	15	150	6.86
DLPFC_L	−15	51	27	82	5.75
ACC_L	−9	45	15	118	4.64
post-rTMS < pre-rTMS					
MOG_L	−21	−81	12	140	−4.58
SOG_L	−21	−87	27	66	−2.22

Differences in ReHo and ALFF between pre-rTMS and post-rTMS in adolescents with MDD (paired-sample *t*-tests, *p* < 0.05, AlphaSim corrected; combined height threshold *p* < 0.05, and minimum cluster size 925 voxels for ReHo and 508 voxels for ALFF). mPFC, medial prefrontal cortex; mOFC, medial orbital frontal cortex; ACC, anterior cingulate gyrus; CUN, cuneus; MCC, middle cingulate cortex; CAL, calcarine; DLPFC, dorsal lateral prefrontal cortex; MOG, middle occipital gyrus; SOG, superior occipital gyrus.

**Table 3:** Comparison of sgACC-based FC between pre-rTMS and post-rTMS.

Brain Region	MNI Coordinates			Cluster Size	T-Value
	x	y	z	(Voxels)	
post-rTMS > pre-rTMS					
DLPFC_L	−46	38	30	172	10.31
IFGoper_R	51	9	27	75	6.48
MCC_L	−3	−3	45	44	16.61
MFG_R	45	12	42	59	5.38
PCUN_L	−15	−57	60	105	3.23
PoCG_L	−18	−42	75	67	4.04
PoCG_R	36	−24	45	54	3.47
SMA_L	−9	−12	78	105	6.6
SMG_L	−51	−36	27	229	13.86

The longitudinal comparisons revealed that the sgACC-based FC in adolescents with MDD was increased after rTMS treatment (paired-sample *t*-tests, *p* < 0.05, AlphaSim corrected; combined height threshold *p* < 0.05, and minimum cluster size 453 voxels). DLPFC, dorsal lateral prefrontal cortex; IFGoper, inferior frontal gyrus, opercular part; MCC, middle cingulate cortex; PCUN, precuneus; MFG, middle frontal gyrus; PoCG, post-central gyrus; SMA, supplementary motor area; SMG, superior marginal gyrus.

## Data Availability

The data presented in this study are available upon reasonable request from the corresponding author.
